# Monovalent antibody-conjugated lipid-polymer nanohybrids for active targeting to desmoglein 3 of keratinocytes to attenuate psoriasiform inflammation

**DOI:** 10.7150/thno.56995

**Published:** 2021-03-04

**Authors:** Zih-Chan Lin, Tsong-Long Hwang, Tse-Hung Huang, Kohei Tahara, Jiří Trousil, Jia-You Fang

**Affiliations:** 1 Graduate Institute of Biomedical Sciences, Chang Gung University, Kweishan, Taoyuan, Taiwan.; 2 Graduate Institute of Natural Products, Chang Gung University, Kweishan, Taoyuan, Taiwan.; 3 Chinese Herbal Medicine Research Team, Healthy Aging Research Center, Chang Gung University, Kweishan, Taoyuan, Taiwan.; 4 Research Center for Food and Cosmetic Safety and Research Center for Chinese Herbal Medicine, Chang Gung University of Science and Technology, Kweishan, Taoyuan, Taiwan.; 5 Department of Chemical Engineering, Ming Chi University of Technology, New Taipei City, Taiwan.; 6 Department of Anesthesiology, Chang Gung Memorial Hospital, Kweishan, Taoyuan, Taiwan.; 7 Department of Traditional Chinese Medicine, Chang Gung Memorial Hospital, Keelung, Taiwan.; 8 School of Traditional Chinese Medicine, Chang Gung University, Kweishan, Taoyuan, Taiwan.; 9 School of Nursing, National Taipei University of Nursing and Health Sciences, Taipei, Taiwan.; 10 Laboratory of Pharmaceutical Engineering, Gifu Pharmaceutical University, Gifu, Japan.; 11 Institute of Macromolecular Chemistry, Czech Academy of Sciences, Prague, Czech Republic.

**Keywords:** desmoglein 3, keratinocyte, psoriasis, lipid-polymer nanohybrid, active targeting, monovalent antibody

## Abstract

To improve the treatment of psoriasiform inflammation, we developed actively targeted nanocarriers loaded with the phosphodiesterase 4 inhibitor AN2728.

**Methods:** Phospholipid-poly(lactic-*co*-glycolic acid) nanohybrids were prepared and conjugated with monovalent anti-desmoglein 3 antibody to bind keratinocytes.

**Results:** The actively targeted nanohybrids were 229 nm in mean size with a nearly neutral surface charge. Flow cytometry and confocal microscopy showed a 9-fold increase in keratinocyte uptake of targeted nanohybrids relative to non-targeted nanoparticles. The nanoparticles localized mainly in lysosomes after internalization. AN2728-loaded antibody-conjugated nanocarriers inhibited cytokine/chemokine overexpression in activated keratinocytes without affecting cell viability. The targeted nanohybrids also suppressed neutrophil migration by reducing CXCL1 and CXCL2 release from keratinocytes. Following subcutaneous administration in mice, the nanohybrids distributed to the epidermis and hair follicles. In a psoriasis-like skin mouse model, the actively targeted nanoparticles were superior to free drug and non-targeted nanoparticles in mitigating skin inflammation. Intervention with the targeted nanosystem reduced the epidermal thickness of the psoriasiform lesion from 191 to 42 µm, decreased the Psoriasis Area Severity Index by 74%, restored barrier function, and returned chemokine levels to baseline.

**Conclusions:** Our developed nanosystem was safe and demonstrated efficient targeting properties for the treatment of cutaneous inflammation.

## Introduction

Psoriasis is an autoimmune skin inflammation involving interactions between keratinocytes and immune cells. Psoriasis is characterized by keratinocyte proliferation and immune cell accumulation in epidermis/dermis 1. The clinical observation includes erythematous papules with white multilayered scales and thickened acanthotic epidermis. While the global prevalence of psoriasis is about 2-3%, its incidence seems to be increasing 2. Keratinocytes demonstrate a critical capacity to initiate psoriatic inflammation by activating the onset of the pathogenic event and sustaining the prolonged phase 3. Long-term therapies incompletely resolve psoriasis because of their inefficiency after prolonged application and side effects 4. Therefore, new treatment strategies are needed to improve therapy.

Loading antipsoriatic drugs into nanocarriers is one approach to improve their therapeutic efficiency. The nanoparticles protect the drugs from degradation and provide controlled and targeted delivery, leading to improved therapy, reduced dose, and minimized adverse effects 5. Active targeting of nanoparticles to target cells is possible by furnishing the nanoparticle surface with specific ligands for receptors on the cell membrane. Antibody-based targeting is promising for active targeting due to its high specificity 6. Increasing applications of monoclonal antibodies in the targeted treatment of psoriasis also encourages the potential of antibody-conjugated nanocarriers. Desmoglein 3 (Dsg3) is a desmosomal glycoprotein that provides calcium-dependent adhesive integrity among keratinocytes 7. Anti-Dsg monoclonal antibody has been proven to specifically target keratinocytes 8, 9. Since Dsg3 is overexpressed in keratinocytes, it is an appropriate target for mitigating psoriatic inflammation with minimum effects on normal tissue.

There are very few actively targeted antibody-conjugated nanosystems for treating cutaneous inflammation. To improve psoriasis management, we developed Dsg3 antibody-conjugated lipid-polymer hybrid nanoparticles encapsulating AN2728. The model drug AN2728 (crisaborole) is a phosphodiesterase 4 (PDE4) inhibitor approved by the USFDA for atopic dermatitis treatment 10 and is also successful in relieving psoriasis 11. PDE4 inhibitors limit the breakdown of cyclic adenosine monophosphate (cAMP) to decrease the levels of proinflammatory cytokines and chemokines 12. Apremilast is an oral PDE4 inhibitor that was approved for psoriasis treatment in 2014 13. PDE4 inhibitors attenuate inflammation by directly modulating the function of keratinocytes 14. Lipid-polymer nanohybrids were selected as the AN2728 nanocarrier because these hybrid materials combine the advantages of lipid-based and polymer-based nanosystems, including excellent storage stability, easy fabrication, high biocompatibility, facile cell uptake, and high loading capacity of lipophilic drugs 15. Polyethylene glycol (PEG)ylated phospholipid and poly(lactic-*co*-glycolic acid) (PLGA) were used to fabricate the nanocarriers because of their biodegradability and approval by the USFDA 15. To effectively target the nanoparticles to keratinocytes, we used a monovalent anti-Dsg3 antibody fragment with free thiol moieties. Full antibody ligands have a number of disadvantages for nanoparticle conjugation that lead to off-target effects 16. Foremost, the orientation of the antibody on the nanoparticle surface is usually random, which reduces its selective binding ability 17. Further, the large size of whole antibodies causes steric hindrance during receptor targeting. Full antibody ligands are also immunogenic and have poor stability. In comparison, antibody fragments, including fragment antigen-binding (Fab) region, single-chain variable fragment (scFv), and monovalent fragment, retain specific antigen binding and specificity during nanoparticle conjugation 18. Of these fragment types, monovalent fragments involve less complicated preparations. The monovalent antibody used in this study is a half-antibody fragment that has better stability than the full-length antibody 19. We evaluated the effects of our nanosystem on psoriasis mitigation in imiquimod (IMQ)-stimulated keratinocytes and an IMQ-induced psoriasiform lesion mouse model.

## Methods

### Preparation of Dsg3 antibody-conjugated nanoparticles

**Figure 1:**
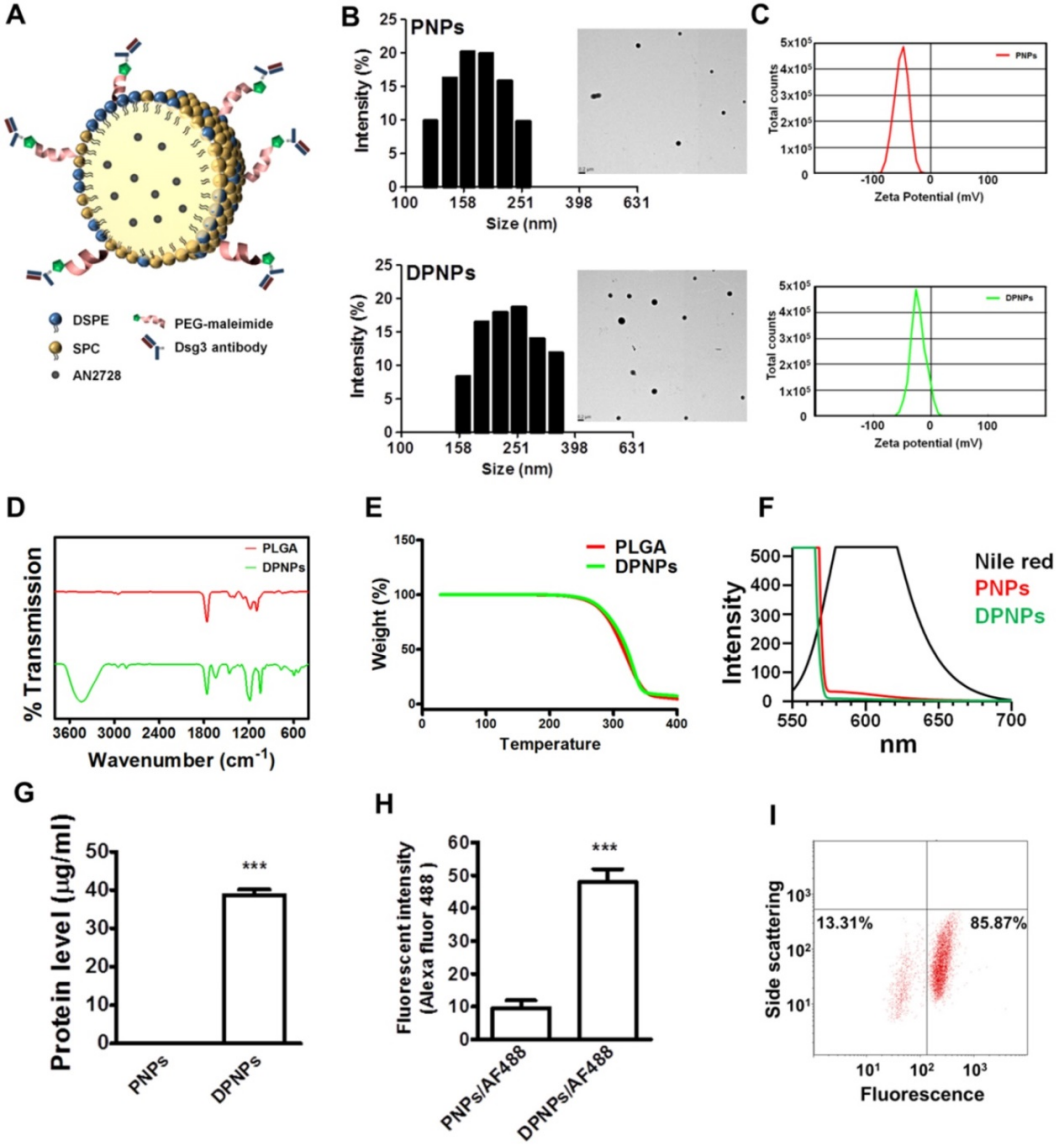
Preparation and characterization of lipid-polymer nanohybrids. (A) Schematic diagram of keratinocyte-targeted hybrid nanoparticles. (B) Dynamic laser scattering and transmission electron microscopy verifying the size and shape (spherical) of the nanoparticles. (C) Zeta potential of PNPs and DPNPs. (D) FTIR spectra recorded in the spectral range of 400~4000 cm^-1^. (E) TGA data for checking thermal stability. (F) Fluorescence emission profiles of Nile red in hybrid nanoparticles. (G) The standard Bradford protein assay demonstrating the efficacy of antibody conjugation on nanoparticles. (H) Antibody immobilization on the hybrid nanoparticle surface. (I) Flow cytometry detection of the binding efficiency of hybrid nanoparticles. Data are expressed as mean ± SEM (*n*=3), ***P < 0.001.

PLGA (50 mg) and AN2728 (2 mg) or rhodamine 800 (0.2 mg) in dichloromethane were injected into 1,2-distearoyl-*sn*-glycero-3-phosphoethanolamine-*N*-[maleimide(polyethylene glycol)-2000] (DSPE-PEG-maleimide, 1 mg), soybean phosphatidylcholine (SPC, 1 mg), and polyvinyl alcohol emulsifier then immediately rigorously emulsified using a high-power sonicator. This resulted in the formation of PLGA nanoparticles encapsulating AN2728 or rhodamine 800. Dsg3 antibody (50 μL, 1 mg/mL, Invitrogen) was reduced to a monovalent fragment by incubating the antibody in 7.5 mM 2-mercaptoethylamine (2ME) in HEPES buffer at 37 °C for 1 h 20. The nanoparticles were incubated with the monovalent antibody for 4 h at room temperature to fabricate Dsg3 antibody-conjugated nanoparticles (DPNPs). Four types of nanoparticles were fabricated with the following compositions from core to outer surface: (i) PLGA matrix, (ii) SPC-decorated surface, (iii) DSPE-PEG-maleimide cross-linker intercalated in the SPC surface, and (iv) anti-Dsg3 antibody conjugated to DSPE-PEG-maleimide (DPNPs). The proposed structure of DPNPs loaded with AN2728 is illustrated in Figure 1A. The nanoparticles (iii) without antibody conjugation were named PNPs. We removed the solvent by evaporation on a magnetic stirrer for 24 h and purified AN2728- or rhodamine 800-loaded nanoparticles by ultracentrifugation (12,000 ×*g*) for 30 min followed by washing thrice and resuspending in phosphate-buffered saline (PBS). The nanodispersions were centrifuged at 48,000 ×*g* and 4 °C for 30 min to withdraw unencapsulated AN2728 in the supernatant. The nanoparticle pellet was reconstituted in water to achieve the desired concentration of AN2728. This procedure ensured complete entrapment of AN2728 or rhodamine 800 in the nanohybrids for further experiments.

### Nanoparticle size measurement

Dynamic light scattering (DLS, Malvern Nano ZS90) was used to measure the particle size distribution, polydispersity index (PDI), and zeta potential of the various nanoparticles. The z-average was calculated as the intensity-based average size from a specific fit to the light scattering correlation function data. The mean of the particle distribution is the z-average and the width determines the PDI.

### Nanoparticle morphology assessment

The morphology of the nanoparticles was visualized by transmission electron microscopy (TEM). A drop of the nanodispersion was deposited onto a grid to form a thin-film without staining. Images were obtained at 200 kV on an ultra-high-resolution TEM (Hitachi HT7800).

### Surface functional group assessment

Fourier transform infrared spectroscopy (FTIR, RX1, Perkin-Elmer) was applied to confirm antibody conjugation on DPNPs and the absence of surface functional groups on PNPs. All experiments were performed at room temperature. The spectral region between 4000 and 400 cm^-1^ was scanned and the spectra were recorded using the KBr disc method.

### Thermogravimetric analysis

Thermal stability of the nanoparticles was determined by thermogravimetric analysis (TGA) using a TA Instrument TGA 2050 thermogravimetric analyzer under nitrogen atmosphere from 25 to 700 °C.

### Antibody conjugation efficacy

PNPs and DPNPs were resuspended in equal amounts of ddH_2_O and then protein content was quantified using a Bradford assay. The samples were incubated at room temperature for 10 min, and then absorbance was measured at 590 nm in an enzyme-linked immunosorbent assay (ELISA). To determine the extent of anti-Dsg3 antibody immobilization, DPNPs were incubated with Alexa Fluor 488-conjugated goat anti-rabbit IgG secondary antibody (Invitrogen) for 1 h at room temperature. After centrifugation, unbound secondary antibody was removed, and the fluorescence of the conjugated secondary antibody was measured using a fluorescence spectrophotometer (Hitachi F2500) and flow cytometer (BD Biosciences FACSCalibur).

### Drug encapsulation efficiency

Nanoparticle precipitate was obtained by ultracentrifugation at 12,000 ×*g* for 30 min at 4 °C. The nanoparticle precipitate was dissolved directly in acetonitrile and the amount of AN2728 in the solution was measured by HPLC. A stainless steel C18 column (25 cm long, 4 mm inner diameter, Merck LiChrospher) was used as the stationary phase. The mobile phase consisted of acetonitrile and ammonium dihydrogen phosphate buffer (65:35). The encapsulated drug was detected by absorption at 282 nm to measure the entrapment efficiency.

### Cell culture

Immortalized human keratinocytes (HaCaT) were obtained from AddexBio and human foreskin fibroblast Hs68 cells were obtained from Bioresource Collection and Research Center. The cells were routinely cultured in Dulbecco's modified eagle medium (DMEM) supplemented with 10% fetal bovine serum and 1% antibiotic-antimycotic in an atmosphere of 5% CO_2_ at 37 °C. Cells from passages 5-10 were used for the experiments. To activate an inflammatory condition, the cells were stimulated with IMQ (5 μg/mL) for 24 h.

### Cellular uptake efficiency

The fluorescence of the cells was analyzed by flow cytometry after treating with PNPs and DPNPs containing 1 μM AN2728 for 8 h. The isotype control of DPNPs was nanocarriers decorated with lymphocyte antigen 6 complex locus G6D (Ly6G) antibody. The conjugation method for anti-Ly6G antibody was the same as that described above for anti-Dsg3 antibody. Fluorescence from a gated population of HaCaT cells labeled with rhodamine 800 was acquired on channel FL3 (> 650 nm) with excitation by a 488 nm solid-state laser. Data were collected from at least 10,000 cells at a flow rate of 35 μL/min. A logarithmic scale was used to measure both background and cell fluorescence. Cell internalization of rhodamine 800-labeled nanohybrids was also assessed by confocal fluorescence microscopy (Leica TCS SP8 X AOBS). Cells were labelled with 4′,6-diamidino-2-phenylindole (DAPI) and LysoTracker Green DND-26 according to the manufacturer's instructions (Invitrogen). Images were pseudocolored blue for DAPI, green for LysoTracker, and red for rhodamine 800. The fluorescence intensity of rhodamine 800 was quantified from the captured images using ImageJ software.

### Cytotoxicity assay

HaCaT cells (10^5^ cells/well) were seeded in 96-well plates overnight. The cells were incubated with free AN2728 or AN2728-loaded nanoparticles at various concentrations for 24 h. Then, 3-(4,5-dimethylthiazol-2-yl)-2,5-diphenyltetrazolium bromide (MTT) was added to a final concentration of 0.5 mg/mL, and the plates were incubated for 4 h at 37 °C in a 5% CO_2_ incubator. The cellular MTT was resolved with DMSO. Absorbance at 550 nm was measured using a spectrophotometer.

### Enzyme-linked immunosorbent assay (ELISA)

The protein expressions of various cytokines and chemokines were measured in HaCaT-conditioned medium following incubation with nanohybrids encapsulating AN2728 (1 μM) for 24 h. Samples were also collected from the back skin tissue of mice exposed to nanohybrids following the methods described below. The levels of cytokines (IL-1β, IL-6, IL-17A, IL-17E, IL-22, IL-23, IFN-γ, TNF-α), and chemokines (IL-8, CXCL1, CXCL2) were measured using commercial kits (BioLegend) following the manufacturer's instructions.

### Chemotaxis assay

A chemotaxis assay was carried out in 24-chamber Transwell plates with a pore size of 3 μm. Neutrophils (2.5 × 10^6^ cells/well) were separated from blood samples of healthy volunteers using a protocol approved by the Institutional Review Board at Chang Gung Memorial Hospital, Taiwan (approval number: 201701925B0). The volunteers provided written informed consent to participate. The procedure for neutrophil purification was previously reported 21. Neutrophils were added to the upper wells and conditioned medium harvested from IMQ-stimulated HaCaT cells (DMEM supplemented with 0.25% bovine serum albumin (BSA)) pretreated with free AN2728 (1 μM) or AN2728-loaded nanocarriers was added to the lower wells. The plates were stored at 37 °C for 1.5 h and then placed on ice. Then, 0.5 M EDTA (100 μL) was pipetted into each well and incubated for 10 min. The well insert was removed, and the cell suspension was collected for washing. The cells were counted using an automated cell counter kit (Moxi Z, Orflo).

### Immunoblotting

HaCaT cells (1 × 10^5^ cells/well) were seeded onto 12-well plates and serum starved for 24 h. Then, the cells were incubated with IMQ (5 mg/mL) in the presence or absence of free AN2728 (1 μM) or AN2728-loaded nanoparticles. The cells were collected and centrifuged at 400 ×*g* for 5 min at 4 °C. After probe sonication, the nuclear proteins were obtained by centrifugation at 8000 ×*g* and 4 °C for 10 min. The final supernatant represented the nuclear protein fraction. The protein concentration was estimated using Protein Assay Dye Reagent (Bio-Rad). The proteins were analyzed by SDS-PAGE and transferred onto polyvinylidene difluoride membranes. The membranes were incubated with phospho (p)-NF-κB or anti-lamin B1 antibody (1:1000, Abcam) overnight at 4 °C. Then, secondary anti-rabbit or anti-mouse horseradish peroxidase antibody (1:2000, Abcam) was added and incubated for 1 h at room temperature, and the bands were visualized using enhanced chemiluminescence reagent.

### Immunocytochemistry

HaCaT cells were grown on coverslips at a density of 1 × 10^5^ cells/mL, treated with the indicated treatments, then fixed in 4% paraformaldehyde for 30 min. Cells were permeabilized for 15 min at room temperature with PBS containing 0.2% Triton X-100 and then blocked with 2% BSA for 1 h. The coverslips were then incubated overnight with NF-κB p65 primary antibody (1:200 dilution, GeneTex) at 4 °C, then treated with goat anti-rabbit IgG secondary antibody conjugated with Alexa Fluor 488 (1:1000 dilution, Invitrogen) for 1 h. The coverslips were mounted onto glass slides using Slowfade^®^ Gold Antifade Mountant with DAPI and examined by confocal laser scanning microscopy (Leica TCS SP8 X AOBS).

### *In vivo* toxicity assay

Male BALB/c mice (8 weeks old) were used to assay the possible *in vivo* toxicity of the nanohybrids. Free AN2728, AN2728-loaded PNPs and DPNPs, and monovalent Dsg3 antibody (2.5 μg) in 100 μL PBS was subcutaneously injected into the backs of mice (n = 6) every other day for 5 days (3 treatments). The concentration of AN2728 was 5 mg/mL for free form, PNPs, and DPNPs. The control group received PBS only (100 μL). The skin was examined for its gross and microscopic appearances, transepidermal water loss (TEWL), skin surface pH, erythema quantification (a*), and hematoxylin and eosin (H&E) histology. TEWL, pH, and a* were determined using a skin physiological evaluation system (MPA580, Courage and Khazaka) with probes positioned onto the treated skin region. The animals were sacrificed by overdose of isoflurane on day 6 for histology. Skin specimens were fixed in 10% neutral buffered formalin for 24 h, then embedded in paraffin wax, sliced (5 μm thickness), and stained with H&E. Hepatic and renal functions were tested to evaluate any possible toxicity of the injected nanoparticles. Plasma was collected from the tail vein before sacrifice. Glutamic oxaloacetic transaminase (GOT), glutamic pyruvic transaminase (GPT), creatinine (CRE) activity, and blood urea nitrogen (BUN) in the plasma were measured using an automated clinical chemistry analyzer (Fuji Dry-Chem 4000i) following the manufacturer's instructions.

### IMQ-induced psoriasis-like skin inflammation model

Psoriasiform skin was induced in the backs of male BALB/c mice (8 weeks old) according to a protocol modified from van der Fits et al. 22. The animals were categorized into five groups (non-treatment control, IMQ stimulation, free AN2728/IMQ, PNPs/IMQ, and DPNPs/IMQ) with six mice in each group. The dorsal skin of all mice including the control group was shaved. The mice received a daily topical dose of 62.5 mg 5% IMQ cream (Aldara^®^, 3 M Health Care) on their shaved backs for five consecutive days (days 1-5). Mice in the treatment groups were subcutaneously injected in the back with 100 μL free AN2728, PNPs, or DPNPs encapsulated with 5 mg/mL AN2728 on days 1, 3, and 5. Mice in the control group were injected with 100 μL double-distilled water. The mice were sacrificed on day 6 by isoflurane overdose. Psoriasis Area and Severity Index (PASI) was objectively scored as described previously 23. Erythema, scaling, and thickness were recorded independently as a score from 0 to 4. Samples of the dorsal skin were taken for protein extraction and histological visualization. Skin was extracted following a previously described method 24. For Western blotting of Dsg3 and p-NF-κB, the skin extract was transferred to a nitrocellulose membrane and probed with primary antibodies against Dsg3, NF-κB, and GAPDH overnight at 4 °C. The membrane was washed three times and then incubated with anti-mouse horseradish peroxidase antibody (Abcam) for 1 h. The bound antibody was observed by enhanced chemiluminescence reagent.

### Immunohistochemistry (IHC)

Dorsal skin samples were fixed overnight in 10% neutral buffered formalin, embedded in paraffin, and sectioned. After dewaxing and rehydration, the paraffin-embedded sections were subjected to heat-induced epitope retrieval using Bond Epitope Retrieval Solution 2 (Leica Biosystems), according to the manufacturer's instructions, then blocked with diluted normal serum. The sections were incubated with rabbit polyclonal anti-mouse IL-17 (MyBioSource MBS2026152), CXCL1 (Invitrogen PA5-86508), CXCL2 (Invitrogen 701126), Ki67 (Abcam ab15580), Ly6G (Abcam ab238132), or myeloperoxidase (MPO, Abcam ab139748) antibody for 1 h at room temperature (1:1000 dilution), washed with saline containing 0.5% Tween 20, and subsequently incubated at ambient temperature with biotinylated donkey anti-rabbit IgG (Jackson ImmunoResearch Laboratories) for 20 min. Color reaction was visualized using an avidin-biotin complex kit (Vectastain Elite, Vector Laboratories). Photomicrographs were obtained using a digital color camera for microscopy (Olympus DP72). To calculate the neutrophil microabscess number in the epidermis, five areas in three independent experimental tissue sections were analyzed with cellSens software (Olympus Life Science).

### Statistical analysis

All data were analyzed using GraphPad Prism 5 software. Data are expressed as mean ± SEM. Dual comparisons were made with an unpaired Student's *t*-test. Groups were analyzed by ANOVA with Tukey or Dunnett posttests.

### Ethics

All animal experiments were conducted in strict accordance with the Guidelines for the Institutional Animal Care and Use Committee of Chang Gung University (approval number: CGU107-211) and complied with Directive 86/109/EEC from the European Commission.

## Results

### Nanocarrier fabrication and antibody conjugation

A PLGA core was prepared by emulsion-solvent evaporation and then coated with SPC and DSPE-PEG-maleimide to form lipid-polymer nanohybrids. Full-length anti-Dsg3 antibody was divided into two monovalent fragments using 2ME. The SDS-PAGE profile showed a band for the full-length antibody with a molecular weight of ~150 kDa (Figure S1). The band representing half-fragment monovalent antibody was more mobile in the electric field than the full-length antibody, indicating a successful fragmentation. Monovalent anti-Dsg3 antibody was immobilized to PEG crosslinker on the nanohybrid surface by maleimide-thiol conjugation to generate DPNPs.

The size and morphology of the nanosystems were characterized by DLS and TEM. Bare PLGA nanoparticles were determined to have an average size of 220 nm by DLS (Table 1), which reduced to 163 nm after SPC decoration because of the emulsifying capability of the phospholipids. SPC-coated nanoparticles with DSPE-PEG-maleimide crosslinker but without antibody conjugation had an average size of 191 nm. This diameter increased to 229 nm upon antibody immobilization (DPNPs). Both PNPs and DPNPs showed a monomodal distribution (Figure 1B). All nanosystems had a PDI of <0.3, suggesting a narrow particle distribution. The TEM micrographs in Fig. 1b show that the nanoparticles are discrete, spherical structures. The zeta potentials of all nanoformulations were negative, with bare PLGA nanoparticles having the largest negative charge (Table 1). Antibody conjugation significantly shielded the negative charge to near zero (Figure 1C).

The impact of antibody conjugation on the properties of the nanoparticles was further assessed by FTIR, TGA, and polarity measurements. The FTIR spectrum for bare PLGA nanoparticles is shown in Figure 1D and is similar to results from previous studies 25, 26. After antibody conjugation, a peak appeared at 1600 cm^-1^ to 1730 cm^-1^ from the carbonyl groups of DSPE-PEG-maleimide and C-S bonding was observed at 500 cm^-1^ to 600 cm^-1^. Also, a strong and wide peak was observed at 3467 cm^-1^, which is correlated to the amine and amide groups of the antibody 27, 28. We then examined the thermal stability of the hybrid nanocarriers by TGA. There was no significant change in thermal stability after antibody conjugation (Figure 1E). Antibody conjugation was further assessed by a polarity assay. The emission spectra of Nile red in acetone, PNPs, and DPNPs are presented in Figure 1F. Nile red fluorescence is quenched in environments with low lipophilicity. The nanoparticles provided a more hydrophilic environment for Nile red than acetone. Nile red emission in DPNPs was weaker than in PNPs, indicating an increase in hydrophilicity after antibody conjugation.

Successful conjugation of the monovalent antibody to the nanohybrids was demonstrated by protein quantification (Figure 1G). To further verify the conjugation, the antibody fragment was labeled with an Alexa Fluor 488-labelled secondary antibody and the fluorescence intensity was measured (Figure 1H). Immobilization of the monovalent antibodies on DPNPs led to a 5-fold increase in Alexa Fluor 488 emission compared to PNPs. Nearly 86% of DPNPs were successfully conjugated with anti-Dsg3 antibody, as measured by flow cytometry (Figure 1I).

### Anti-Dsg3 antibody conjugation enhances keratinocyte uptake of the nanohybrids

**Figure 2:**
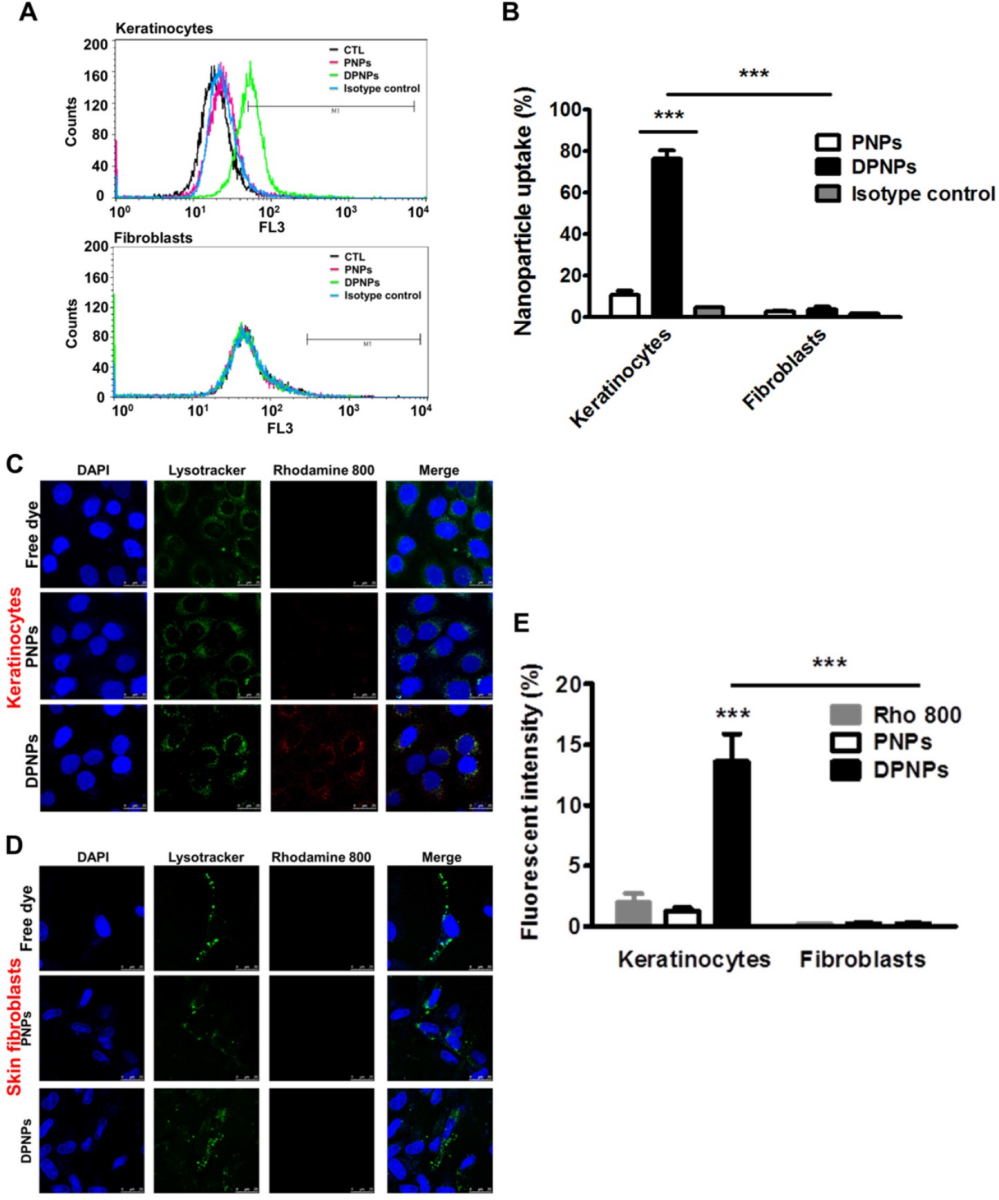
*In vitro* targeting efficiency of lipid-polymer nanohybrids. (A) Representative flow cytometry histogram of rhodamine 800-labeled hybrid nanoparticles uptake in human keratinocytes and fibroblasts over a period of 8 h. (B) Percentage of the cell population for human keratinocytes and fibroblasts that has internalized rhodamine 800-labeled hybrid nanoparticles. (C, D) Overlay of DAPI (blue), lysotracker (green), and rhodamine 800 (red) in fluorescence microscopic images showing uptake of rhodamine 800-labeled DPNPs in keratinocytes (HaCaT) and fibroblasts (Hs68) over a period of 8 h. Free rhodamine 800 solution was served as a control. Scale bars, 25 µm. (E) The fluorescence intensity was plotted graphically. Data are expressed as mean ± SEM (*n*=4). ***P < 0.001, compared to control or free dye. The control is the HaCaT cells treated by PBS only.

To confirm the presence of Dsg3 on keratinocyte membrane, keratinocytes were treated with full-length anti-Dsg3 antibody conjugated with Alexa Fluor 488. Confocal imaging results reveal abundant Dsg3 on the cell membrane of non-treated and IMQ-treated keratinocytes (Figure S2). Next, a cell dissociation assay was conducted to examine if the anti-Dsg3 antibody affects cell-cell adhesion. Both full-length and monovalent antibody treatments maintained keratinocyte aggregation (Figure S3). Therefore, the antibody is not bioactive and is not involved in signal transduction. Keratinocyte uptake of rhodamine 800-loaded PNPs and DPNPs was assessed for 8 h by flow cytometry and confocal microscopy. The flow cytometry results show a dramatic signal shift for DPNPs compared to PNPs (Figure 2A), indicating the specificity of the monovalent antibody to Dsg3. The isotype control nanoparticles showed no signal shift when compared to PNPs, suggesting that only the Dsg3 antibody was capable of binding the keratinocyte membrane. The fluorescence histogram of Dsg3-negative skin fibroblasts incubated with DPNPs was similar to that of fibroblasts incubated with PNPs. Further, the fluorescence intensity measured by flow cytometry was 6-fold higher in keratinocytes treated with DPNPs than PNPs (Figure 2B). The uptake of nanohybrids in keratinocytes was also parallelly analyzed by confocal image (Figure 2C). Intense fluorescence from Alexa Fluor 488 (pseudocolored red) was detected in the cytoplasm of keratinocytes treated with DPNPs but not PNPs. The morphology of the nuclei (blue) remained intact after treatment. Punctate DPNPs signal was colocalized with lysosomes (green). No red fluorescence was observed in the skin fibroblasts incubated with DPNPs (Figure 2D), suggesting the selective targeting of DPNPs to keratinocytes but not fibroblasts. The fluorescent intensity estimated from the confocal image shows 9-fold stronger uptake of DPNPs in HaCaT cells than of PNPs (Figure 2E). Therefore, the anti-Dsg3 antibody fragment exhibits the potential as a ligand to enhance internalization of the nanohybrids in keratinocytes.

### Actively targeted nanohybrids effectively suppress keratinocyte stimulation

**Figure 3:**
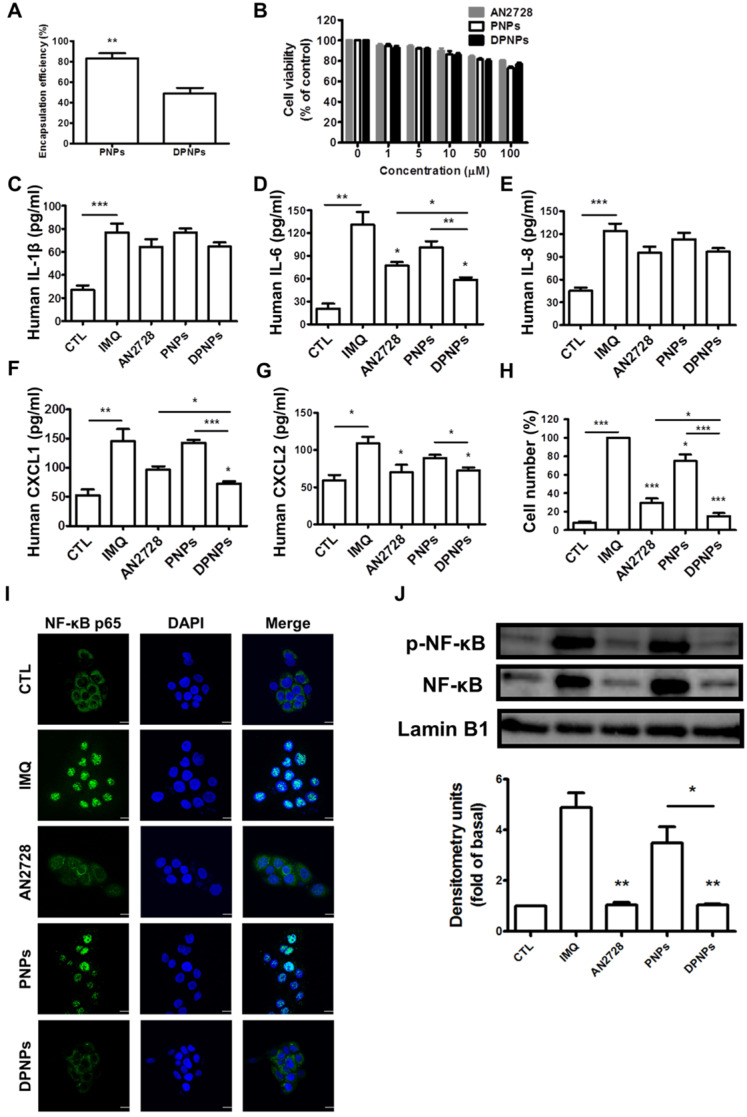
The effect of the AN2728-loaded lipid-polymer nanohybrids on cytokine and chemokine expression in IMQ-induced HaCaT keratinocytes. (A) The encapsulation efficiency of nanoparticles. (B) HaCaT keratinocytes were treated with various concentrations of different AN2728-loaded nanoparticles for 24 h. The cell viability of the cells was determined by MTT assay. (C-G) HaCaT keratinocytes were pretreated with free AN2728, PNPs, or DPNPs for 4 h before the addition of IMQ for 24 h, after which, cytokines (C) IL-1β, (D) IL-6 and chemokine (E) IL-8, (F) CXCL1, (G) CXCL2 protein expression was examined using ELISA. (H) Chemotaxis assay of neutrophils from IMQ-induced HaCaT keratinocyte condition medium pretreated with free AN2728, PNPs, or DPNPs before stimulation with IMQ. (I) Representative immunofluorescence of p-NF-κB in HaCaT cells. The cells were pretreated with different condition for 4 h and then stimulated with IMQ. Scale bars, 25 µm. (J) Representative immunoblot analysis using antibodies against p-NF-κB, and lamin B1. Data are expressed as mean ± SEM (*n*=4). * *P* < 0.05, ** *P* < 0.01, *** P < 0.001, compared with the group of IMQ treatment alone. The statistical difference was also detected between the different treatment groups. The control is the HaCaT cells without IMQ activation.

To test whether PDE4 inhibitor-loaded DPNPs could suppress inflammation in IMQ-stimulated keratinocytes by binding to Dsg3, we first evaluated the AN2728 encapsulation efficiency. The entrapment percentages for PNPs and DPNPs were 83% and 50%, respectively (Figure 3A). The nanosystems were centrifuged to remove non-encapsulated AN2728 in the supernatant, and then the nanocarriers were reconstituted in water to achieve the desired concentrations. Thus, all the drug molecules were loaded in the nanohybrids. Treatment with free drug and drug-loaded nanohybrids exerted a dose-dependent decline in IMQ-stimulated keratinocyte viability within the concentration range of 1-100 μM (Figure 3B). This cytotoxic effect was modest, as >80% viability was maintained with 100 μM AN2728. We observed no significant difference in viability between free drug and nanocarriers treatments. Thus, the non-toxic dose of 1 μM was chosen to explore cell uptake and anti-inflammatory activity. We detected marked increases in the levels of the proinflammatory mediators IL-1β, IL-6, IL-8, CXCL1, and CXCL2 in IMQ-stimulated keratinocytes (Figure 3C-G). All AN2728 formulations inhibited the overexpression of these mediators at the protein level. There was no statistically significant difference in the reductions of IL-1β and IL-8 by free drug and nanocarriers, indicating that not all cytokines could be regulated by AN2728. The general trend in reduction of cytokines/chemokines levels was DPNPs > free AN2728 > PNPs. Further, compared to the other mediator markers, a greater reduction in IL-6 (55%) and CXCL1 (50%) levels was observed in cells treated with DPNPs.

The chemokines released by keratinocytes are vital for prompting neutrophil infiltration and activation. A chemotaxis assay of human neutrophils was carried out to examine their migration toward medium conditioned by IMQ-stimulated keratinocytes treated with free drug or nanocarriers. The greatest inhibition in neutrophil migration number was observed for DPNPs, followed by free drug and PNPs (Figure 3H). The antibody-conjugated nanocarriers completely restrained neutrophil migration to baseline. PDE4 inhibitor participates crucially in the NF-κB signaling pathway. To further elucidate the role of NF-κB in nanohybrid inhibition of activated keratinocytes, we assessed the IMQ-induced nuclear translocation of p-NF-κB and total NF-κB by confocal microscopy (Figure 3I). This translocation was prevented by AN2728 in keratinocytes. DPNPs dramatically retarded the nuclear expression of p-NF-κB, and this effect was superior to that of PNPs. Western blot demonstrated that both free AN2728 and DPNPs almost completely suppressed p-NF-κB and total NF-κB expressions in the nuclei, while this was not the case for PNPs (Figure 3J). These results, therefore, suggest that the anti-Dsg3 antibody promoted the ability of the drug-loaded nanoparticles to reduce keratinocyte activation by enhancing binding to the cells.

### The nanohybrids demonstrate negligible acute toxicity in the skin and other organs

**Figure 4:**
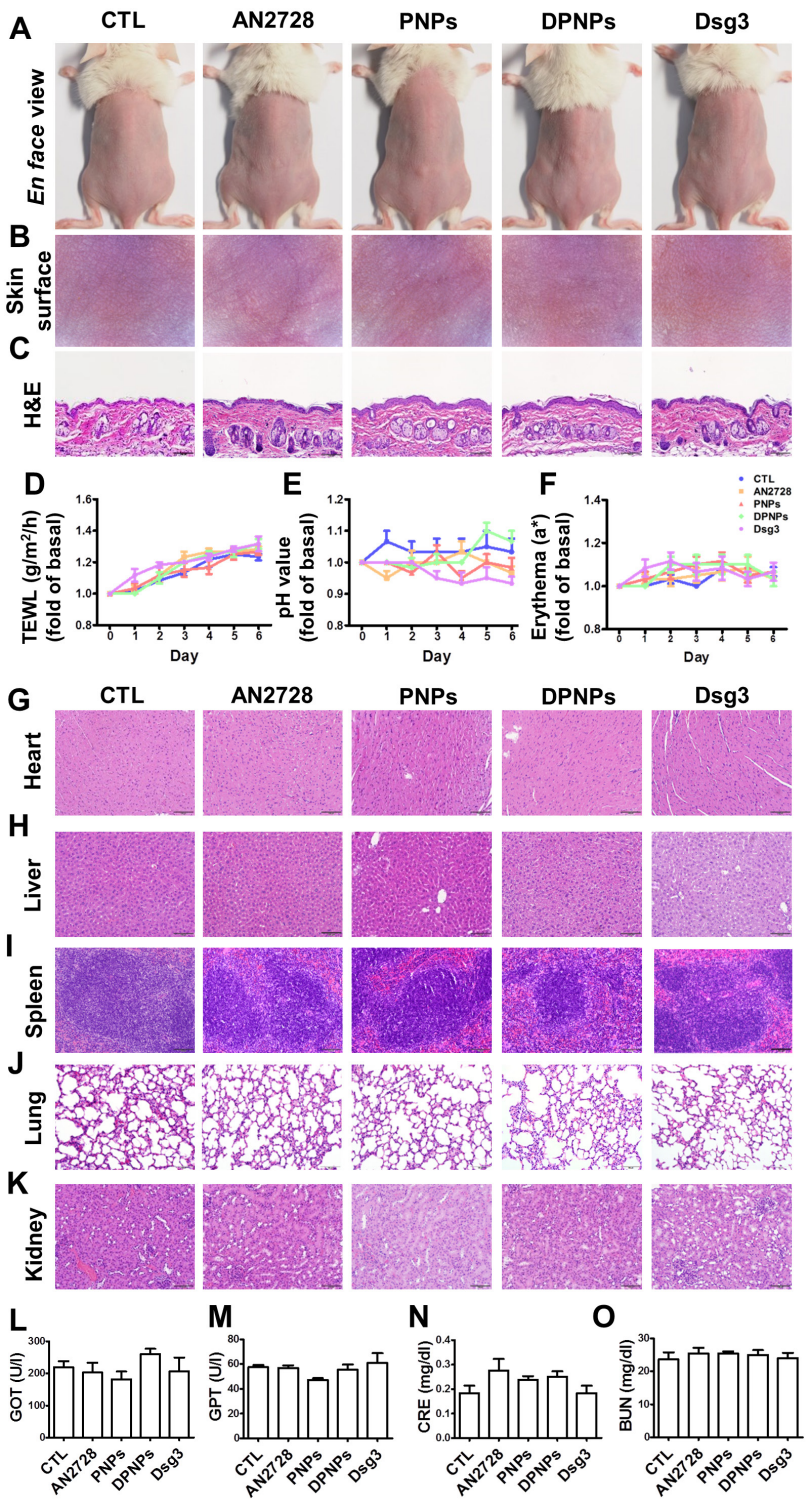
Physiological and histological observations for mice received different administration of AN2728, PNPs, DPNPs, or Dsg3 antibody. Mice were intradermally injected with 100 µl of 1 mg/ml free AN2728 or different nanoparticles or 2.5 µg Dsg3 antibody in PBS on Day 1, 3, and 5. Tissue samples of heart, liver, spleen, lung, kidney, skin, and blood were collected on Day 6: (A) The gross images of mouse back skin were represented on Day 6 by digital camera. (B) The close-up images by hand-held digital microscopy. (C) Skin sections represented by H&E staining. (D) TEWL. (E) skin surface pH value. (F) erythema quantification. H&E staining of (G) heart, (H) liver, (I) spleen, (J) lung, and (K) kidney. Scale bars, 100 µm. Blood biochemical parameters of (L) GOT, (M) GPT, (N) CRE, and (O) BUN. Scale bars, 100 µm. Data are expressed as mean ± SEM (*n*=6).

Administration of nanoparticles or antibodies via a subcutaneous route may evoke skin and systemic side effects. Free drug, PNPs, DPNPs, or free antibody were subcutaneously delivered to healthy mouse skin every two days for five days. The gross and en face appearances of the skin surface did not change after treatment (Figure 4A-B). Histological examination further revealed that the skin morphology remained intact without any significant changes (Figure 4C). During the five days, there was no increase in TEWL, skin pH, or erythema for all the formulations compared with the control (Figure 4D-F). The gradual elevation of TEWL in the control and treated samples over six days could be due to the hydration of epidermis by injection vehicle (water). These results demonstrate that the nanohybrids and antibody were well-tolerated for skin application.

When subcutaneously administered nanoparticles enter the blood or lymphatic vessels, they distribute to the peripheral organs. Histopathological analysis of the major organs including heart, liver, spleen, lung, and kidney reveal no abnormality for all formulations tested (Figure 4G-K). Therefore, these formulations were relatively non-toxic even if they spread to other organs. We also performed blood tests to measure liver and kidney functions (Figure 4L-O). Blood biochemical markers were within normal ranges after treatment. Thus, free AN2728, the nanocarriers, and the monovalent antibody did not inflict any adverse effect over the 5-day treatment.

### Actively targeted nanohybrids effectively mitigate psoriasiform lesions

**Figure 5:**
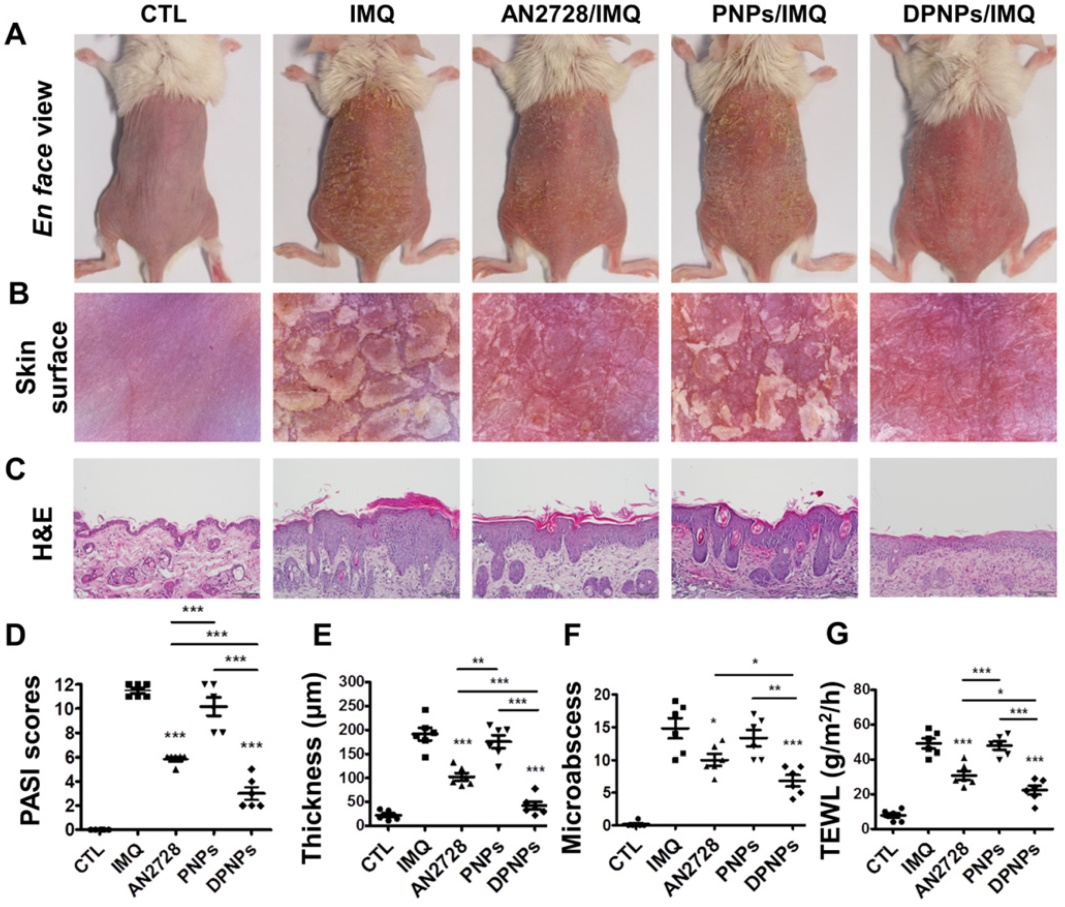
The keratinocyte-targeted lipid-polymer nanohybrids attenuate IMQ-induced mouse psoriatic skin inflammation. The mice were subcutaneously application with different nanoparticles for three times on Day 1, 3, and 5, then the psoriasis-like skin were induced by IMQ cream from Day 1 to Day 5. Mouse back skin was collected on Day 6. (A) The gross images of mouse back skin were represented on Day 6 by digital camera. (B) The close-up images by hand-held digital microscopy. (C) Skin sections were represented by H&E staining. Scale bars, 100 µm. (D) Quantification of mouse skin histology using the PASI score. (E) Epidermal thickness quantified by H&E staining. (F) Total counts of abscesses by Ly6G and MPO IHC staining. (G) TEWL measurement. Data are expressed as mean ± SEM (*n*=6). * *P* < 0.05, ** *P* < 0.01, *** P < 0.001.

As a proof of concept, a psoriasis-like animal model was established by topical IMQ intervention, and the *in vivo* anti-inflammatory activity of the nanohybrids was examined and compared to that of free AN2728. Phenotypic and microscopic images of the IMQ-treated skin surface exhibit white scaling, erythema, and thickening (Figure 5A-B). Subcutaneous injection of free drug partly ameliorated psoriasis-like symptoms. A further improvement was seen in the DPNPs treatment group. H&E-stained skin sections indicate that IMQ induced epidermal hyperplasia with hyperkeratosis, rete ridge elongation, and dermal immune cell infiltration (Figure 5C). Munro's microabscesses in the stratum corneum were also detected after IMQ treatment. DPNPs manifested the most significant attenuation of histological pathology, followed by free drug and PNPs. However, there was still some epidermal thickening and immune cell infiltration in DPNPs-treated psoriasiform skin. Epidermal thickness, desquamation, and erythema were rated and summarized as a PASI score (Figure 5D). The highest total PASI score was obtained for the IMQ treatment. Intervention with DPNPs decreased the PASI score by 74% compared to IMQ only. The PASI scores for the free drug and PNPs groups fell between those of the DPNPs and IMQ only groups, presenting limited therapy of the psoriasiform lesion. Free AN2728 and DPNPs significantly reduced the epidermal thickness from 191 to 103 and 42 μm, respectively (Figure 5E), whereas PNPs did not decrease the IMQ-treated epidermal thickness. IMQ application also led to a marked increase in microabscesses and damaged the barrier function of the skin, as determined by TEWL. Both microabscess number and TEWL were improved by the interventions in the order DPNPs > AN2728 > PNPs (Figure 5F-G).

**Figure 6:**
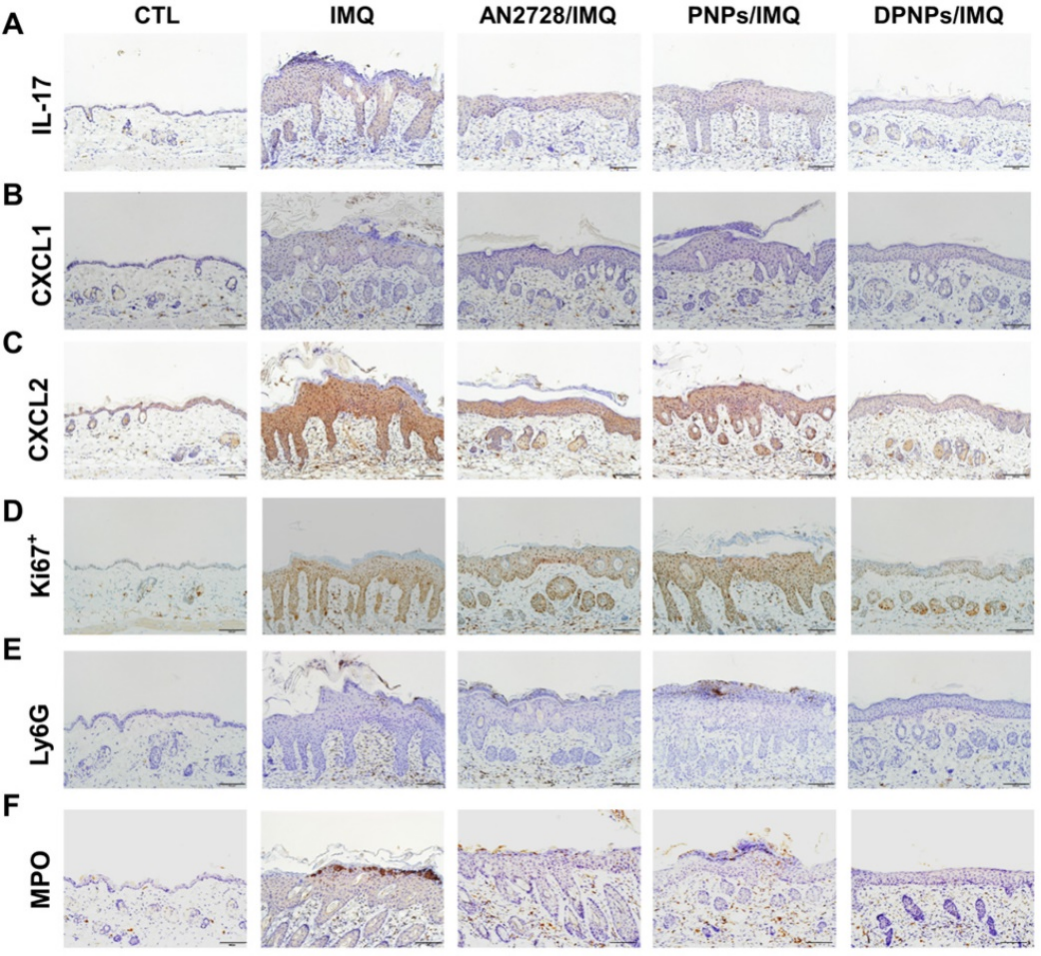
The keratinocyte-target lipid-polymer nanohybrids suppress IMQ-induced cytokine and chemokine expression, as well as neutrophil infiltration in psoriasis-like skin. IHC analysis represents (A) IL-17, (B) CXCL1, (C) CXCL2, (D) Ki67^+^, (E) Ly6G, and (F) MPO in psoriasis-like skin. Scale bars, 100 µm.

Inflammation mitigation by the nanoformulations was further evaluated by IHC. Overexpression of IL-17 in psoriasiform plaques, detected with a pan-IL-17 antibody, was reduced by AN2728 and the nanocarriers (Figure 6A). Similar results were observed for the expressions of chemokines CXCL1 and CXCL2 (Figure 6B-C). Compared to free drug and PNPs, DPNPs further suppressed the level of CXCL2. IHC revealed a much higher Ki67^+^ cell number in the epidermis of IMQ-treated skin than normal control, suggesting there was significant cell proliferation in the plaque (Figure 6D). Keratinocyte proliferation was arrested by DPNPs but not free drug or PNPs. Neutrophil recruitment in the upper epidermis is the primary contribution of Munro's microabscesses. The location of neutrophils in the skin was visualized by Ly6G and MPO (Figure 6E-F). A cluster of neutrophils was found in the upper epidermis. Neutrophil infiltration was also observed in the dermis. Neutrophil accumulation was attenuated in all treated groups, especially by DPNPs.

**Figure 7:**
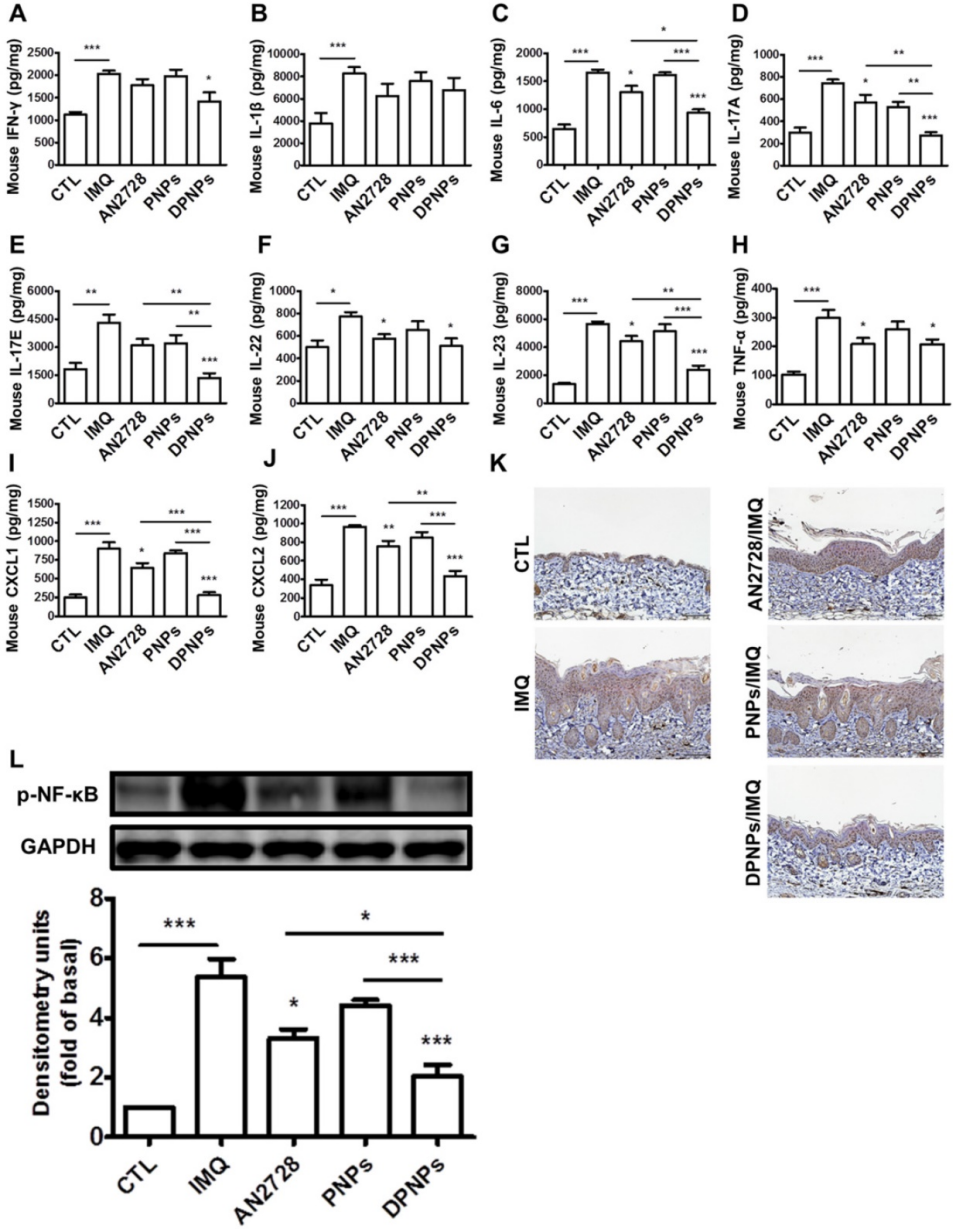
The keratinocyte-targeted lipid-polymer nanohybrids downregulate IMQ-induced NF-κB activation leading to the decrease of cytokine and chemokine expression of psoriasis-like skin. The protein expression of (A) IFN-γ, (B) IL-1β, (C) IL-6, (D) IL-17A, (E) IL-17E, (F) IL-22, (G) IL-23, (H) TNF-α, (I) CXCL1, and (J) CXCL2 in IMQ-induced psoriatic skin. (K) Representative IHC staining for p-NF-κB. Scale bars, 100 µm. (L) Immunoblotting analysis with p-NF-κB. DSC, desmocollin; DP, desmoplakin; PG, plakoglobin; PP, plakophilin. Data are expressed as mean ± SEM (*n*=4). *P* < 0.05, ** *P* < 0.01, *** P < 0.001, compared with control group.

Certain cytokines play essential roles in the development of psoriasis. We detected a series of cytokines in mouse skin (Figure 7A-H). Compared to normal control, the protein levels of cytokines dramatically increased in the skin due to IMQ activation. The mice dosed with free AN2728 had significantly decreased levels of IL-6, IL-17A, IL-22, IL-23, and TNF-α but did not achieve complete attenuation compared to the control. Free AN2728 did not suppress IFN-γ, IL-1β, or IL-17E. A similar result was found for the PNPs-treated group. In comparison, DPNPs inhibited the expressions of IFN-γ, IL-6, IL-17A, IL-17E, and IL-23 to a greater extent and completely inhibited IL-17A and IL-17E expressions to baseline. DPNPs also thoroughly inhibited the chemokines CXCL1 and CXCL2 (Figure 7I-J). NF-κB is an upstream modulator of immune response. Phosphorylation of NF-κB induces cytokines/chemokines to activate psoriatic inflammation. IMQ application on mouse skin significantly increased the p-NF-κB level in the epidermis (Figure 7K), and this overexpression was prevented by DPNPs. This result was further supported by a western blot assay (Figure 7L). p-NF-κB was downregulated 62% by DPNPs compared to IMQ treatment only.

### Actively targeted nanohybrids broadly accumulate in epidermis after subcutaneous administration

**Figure 8:**
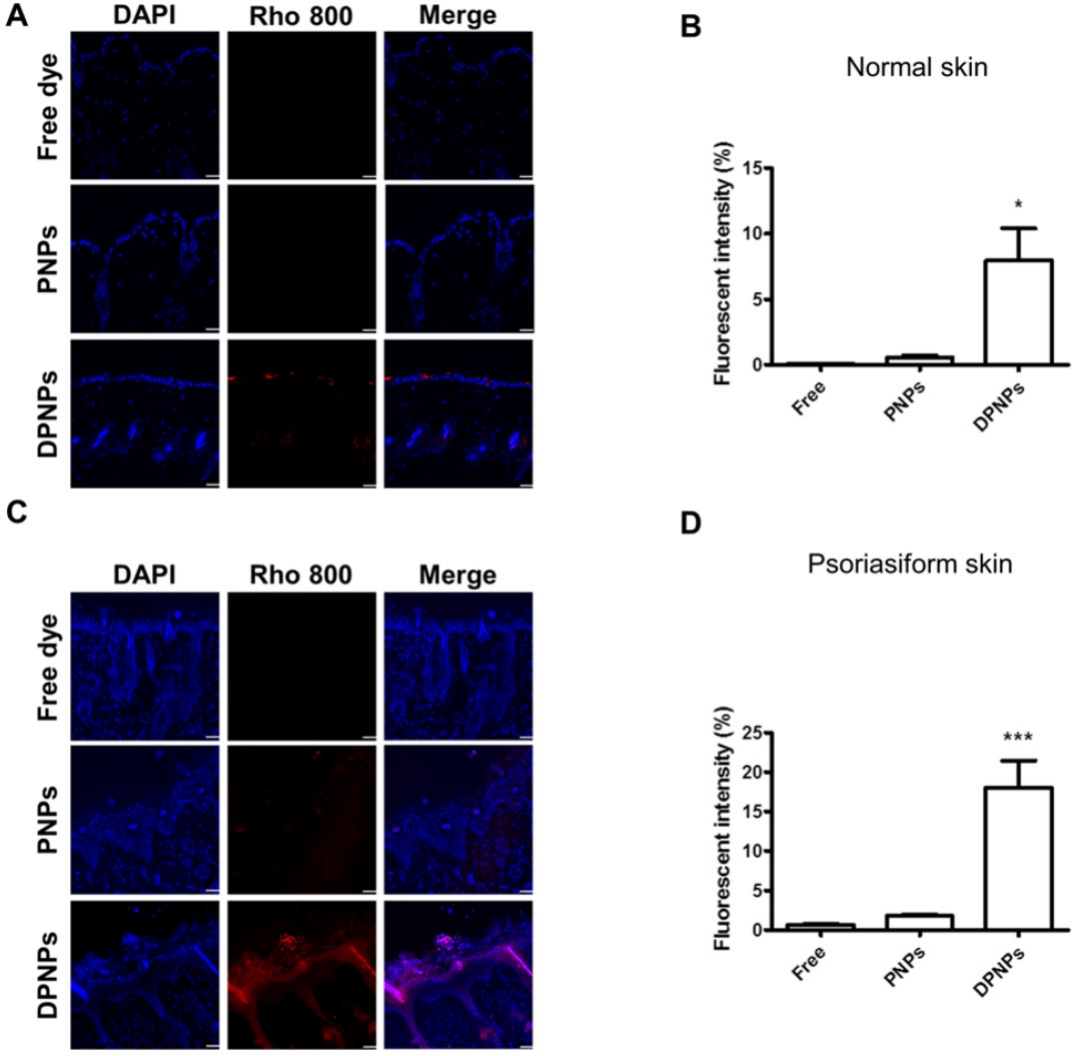
Fluorescent distribution of dye-loaded lipid-polymer nanohybrids in frozen skin section after intradermal injection with free dye, PNPs, or DPNPs into (A) normal mice skin and (C) IMQ-induced psoriasis-like mouse skin by confocal microscopy. Scale bars, 10 µm. (B, D) Quantification of the mean fluorescence intensity of rhodamime 800 by Leica Application Suite X imaging software. Data are expressed as mean ± SEM (*n*=6).

To investigate if the nanocarriers could transit from the dermis to the epidermis and bind to keratinocytes after subcutaneous injection, the distribution of rhodamine 800-loaded nanohybrids in the skin area of the injection site was imaged by confocal microscopy. First, we examined the distribution of Dsg3 in skin. Both healthy and psoriasiform mouse skin showed Dsg3 expression in the epidermis, as observed by confocal microscopy (Figure S4A). Western blotting also revealed higher Dsg3 expression in psoriasiform skin than in normal skin (Figure S4B). No fluorescence signal from rhodamine 800 was observed in normal mouse skin treated with free dye or PNPs (Figure 8A). Cumulative fluorescence was observed in the epidermis and hair follicles of healthy mice treated with rhodamine-loaded DPNPs (Figure 8B). Epidermis thickening was observed in IMQ-treated skin (Figure 8C). While no fluorescence attributed to free dye was observed in psoriasis-like skin, faint fluorescence from PNPs was detected in psoriasiform skin. In comparison, antibody incorporation increased nanoparticle transit through the dermis into the epidermis. DPNPs were specifically and evenly distributed throughout the epidermis and hair follicles. The fluorescence intensity in psoriasis-like skin treated with DPNPs was much higher than that treated with free dye or PNPs (Figure 8D). Cumulatively, these results confirm the efficient targeting of the antibody-conjugated nanohybrids to inflamed tissue. The DPNPs could diffuse to the epidermis from the subcutaneous injection site.

## Discussion

Dsg3 is overexpressed during keratinocyte proliferation 29, 30. We anticipated that decoration of nanocarriers with anti-Dsg3 antibody could enable them to bind to keratinocytes. Therefore, we designed AN2728-loaded phospholipid-PLGA nanohybrids conjugated with a monovalent anti-Dsg3 antibody. This nanosystem effectively suppressed psoriatic inflammation in cell and animal models. Our data demonstrate that this nanosystem prevented keratinocyte activation and psoriasiform deterioration. The nanoparticles were broadly distributed in the epidermis, where they exerted bioactivity. To our knowledge, this study is the first report investigating antibody-conjugated nanohybrids targeting active keratinocytes to treat cutaneous inflammation. PLGA was used as the nanoparticle matrix because it effectively intercalates the cross-linker and antibody. The SPC coating maintained the lipophilicity of the nanoparticle surface for facile uptake and interaction with cells. We utilized DSPE-PEG-maleimide as the conjugation linker to connect the antibody and nanoparticles. PEGylation also improves resistance to enzymatic degradation and protein binding for prolonged residence in the target site 31. DPNPs had an average diameter of 229 nm (Table 1), which is around the ideal size (≤ 200 nm) for polymeric nanoparticles to avoid premature clearance by macrophages 32.

The targeted nanocarriers showed significant internalization by keratinocytes (Figure 2A-B). This result confirmed the conservation of Dsg3 binding activity after reduction of the full antibody to a monovalent antibody fragment. It has been recognized that the cellular uptake efficiency of targeted nanoparticles can be limited in serum because the protein corona masks the targeting ligands 33. In this study, DPNPs were internalized by keratinocytes within 8 h in culture medium containing serum. The cytosolic distribution of the DPNPs indicated that the nanoparticles penetrated across the cell membrane after initially binding to Dsg3. Lysosomal leakage is generally a critical stage in nanoparticle degradation to release encapsulated drug into the cytoplasm 34. Based on confocal imaging of LysoTracker-stained keratinocytes (Figure 2C), we propose that once internalized by keratinocytes, the targeted nanohybrids localized to lysosomes, where they were degraded and their drug cargo was liberated.

Keratinocytes are regarded as the first-line cells of the skin-resident immune system to release proinflammatory mediators for immune cell infiltration in psoriasis 35. A cytotoxicity assay demonstrated that the free drug and drug-loaded nanoformulations did not significantly affect keratinocyte viability (Figure 3B). The nanohybrids also did not cause anti-inflammatory effects on activated keratinocytes. Suppression of keratinocyte activation depends on the intracellular drug dose. Compared with free drug and PNPs, DPNPs better inhibited cytokines/chemokines in keratinocytes (Figure 3C-G) due to their extensive cell uptake via active Dsg3 targeting (Figure 2). Additionally, free AN2728 inhibited cytokines/chemokines more than PNPs. Lipophilic small molecules such as AN2728 can enter cells by passive diffusion when they accumulate outside the cells 36. In comparison, PEGylated PNPs are sterically hindered from entering cells. Stimulated keratinocytes release chemokines during the early phase of psoriasis to recruit neutrophils and dendritic cells in psoriatic plaque 37. PDE4 inhibitors effectively suppress chemokine secretion in keratinocytes in a dose-dependent manner 38. DPNPs better inhibited chemokines than free drug and PNPs (Figure 3F-G), implying that DPNP treatment can best reduce immune cell infiltration. Through chemotaxis analysis, we confirmed significant inhibition of neutrophil migration by DPNPs to nearly baseline (Figure 3H).

Subcutaneous administration of DPNPs dramatically relieved psoriasiform symptoms compared to free drug and PNPs (Figure 5). DPNPs inhibited proinflammatory mediators more than free AN2728, and this difference was more significant in the animal model than the cell-based assay. This result could be due to the reported poor stability and extensive hydrolysis of free AN2728 to produce inactive metabolites 39. Loading active agents into a nanoparticle matrix has been shown to prevent drug leaching and protein or enzymatic attack 40. Although PNPs may also protect loaded AN2728 from degradation, their non-targeted nature led to inefficient delivery and therapy. Additionally, PNPs have a higher surface charge than DPNPs, which may increase clearance by non-targeted immune cells 33. Nanoparticle size is another factor governing immune cell uptake 41. A particle diameter of <300 nm is needed to escape uptake by immune cells 42, and DPNPs fit this criterion.

Dsg3 expression predominantly occurs in the squamous epithelium and hair follicles 43, 44. Subcutaneous nanoparticles must diffuse across the dermis and penetrate tight junctions to reach target keratinocytes. An earlier study demonstrated that 250 nm lipid-based nanoparticles could transport across the keratinocyte monolayer without disturbing the tight junction 45. DPNPs may reach keratinocytes is this way as they are ~250 nm in size. PEG decoration can also improve nanoparticle permeation across the skin by reducing interactions with cellular barriers, biological fluids, and extracellular matrix 46. Although PNPs also possess a PEG coating, their high surface charge and steric hindrance to cellular internalization reduce keratinocyte interactions. We also found accumulation of DPNPs in hair follicles where Dsg3 largely distributes. Keratinocytes are the major cells contributing to the follicular epithelium. The follicular epithelium is an insufficient barrier, so nanoparticles facilely diffuse from the dermis to the follicular canal 47.

Psoriasis involves interplay between keratinocytes and immune cells. The cytokine production in psoriasis relates to the overstimulation of keratinocytes, dendritic cells, neutrophils, T cells, and macrophages 48. *In vivo* treatment with free AN2728 had limited impact on cytokine inhibition. In contrast, targeted delivery of the drug in DPNPs significantly increased inhibition (Figure 7A-H). IMQ-induced inflammation development depends critically on IL-17 and IL-23 49. IL-23 can be released by dendritic cells, T lymphocytes, and macrophages to activate IL-17A-producing T cells, neutrophils, and mast cells 50. Keratinocytes respond to cytokines such as IL-17A by upregulating a range of inflammatory products, including IL-1β, IL-6, IL-17E, and chemokines. IL-17E secreted by keratinocytes destabilizes the epidermal barrier by filaggrin inhibition 51. DPNPs were shown to restrain IL-17E upregulation and restore skin barrier function, as determined by a reduction in TEWL (Figure 5G). The cytokines/chemokines produced by activated keratinocytes stimulate immune cells to expand the inflammatory response. This becomes a vicious circle prompting keratinocyte proliferation in the lesion area 52. For instance, keratinocytes are an important IL-22 target in the skin. IL-22 regulates keratinocyte proliferation to induce epidermal hyperplasia in IMQ-induced inflammation 53. Our data revealed superior suppression of Ki67 expression by DPNPs than other formulations.

**Figure 9:**
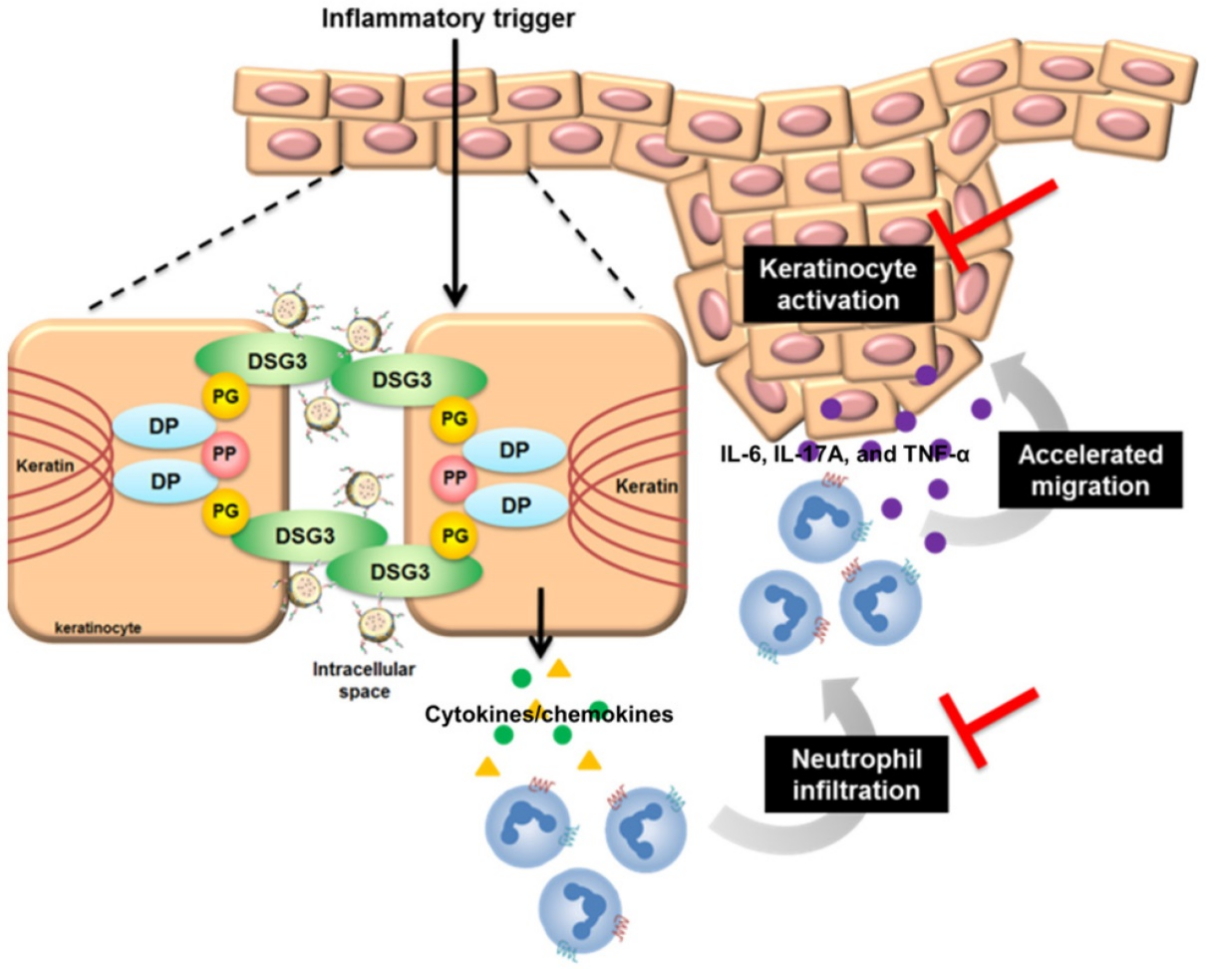
The possible anti-inflammatory mechanisms of DPNPs after internalization into activated keratinocytes.

Keratinocyte-neutrophil interaction characterizes the early pathogenesis of psoriasis 54. Stimulated keratinocytes generate neutrophil-tropic chemokines IL-8, CXCL1, and CXCL2 55. IL-17E from keratinocytes also recruits neutrophils in psoriasis 56. The infiltrated neutrophils produce IL-6, IL-17A, and TNF-α to stimulate keratinocytes, resulting in positive feedback to deteriorate psoriasis. DPNPs inhibited the expressions of IL-17E, CXCL1, and CXCL2 to baseline (Figure 7E, I-J). Thus, the neutrophil infiltration was blocked, stalling the vicious loop. IMQ is known to decrease cAMP and then induce NF-κB phosphorylation 24. This activation enhances expression of IL-1β, IL-6, TNF-α, and chemokines in keratinocytes 57. PDE4 inhibitors elevate cellular cAMP by suppressing NF-κB phosphorylation. It is hypothesized that arresting p-NF-κB would be effective in preventing inflammation. Our results suggest that AN2728-loaded nanohybrids inhibit p-NF-κB translocation from cytoplasm to the nucleus (Figure 3J). DPNPs also significantly reduced the distribution of p-NF-κB in the psoriasis-like lesion (Figure 7L). Therefore, this actively targeted nanosystem potentially inhibited overexpressed cytokines/chemokines via the NF-κB pathway, leading to attenuation of keratinocyte activation and neutrophil recruitment (Figure 9). NF-κB is fundamental for cell viability in multiple organs 58. Its systemic blockade can cause adverse reactions. Our data show that the nanohybrids were well-tolerated in mice with no significant changes in organ histology or biochemical markers following subcutaneous administration. Most studies consider that a PASI reduction of 75% is successful for psoriasis treatment 14. Our DPNPs fulfilled this aim in decreasing the PASI score by 74%. A limitation of this study is the use of a subcutaneous route for delivering the nanohybrids. We used injection to confirm the contact of intact nanocarriers with keratinocytes. In clinical conditions, topical application may be a feasible choice for convenient and noninvasive administration. The nanohybrids still have the opportunity to facilely permeate into the psoriatic skin because of its dysfunctional barrier. Whether topical delivery is beneficial for treating psoriasis remains to be explored. We expected that the subcutaneous DPNPs diffused to epidermis for exerting anti-inflammatory activity. However, we cannot exclude the possibility that DPNPs diffused to circulation or the skin region not in close contact to the injection site for achieving psoriatic lesion targeting. Further pharmacokinetic study is also needed in the future.

## Conclusions

Anti-Dsg3 antibody-conjugated lipid-polymer nanohybrids were successfully fabricated and loaded with a PDE4 inhibitor to improve the therapeutic efficiency of psoriatic inflammation. Uniquely, this nanosystem uses a monovalent antibody with a small size and less complicated preparation than other antibody fragments. The actively targeted nanocarriers had an average size of 229 nm with a neutral surface charge, implying low protein adsorption and minimized immune cell clearance. DPNPs exhibited Dsg3-mediated cell uptake, which contributed to specific targeting to keratinocytes. DPNPs inhibited cytokine/chemokine overexpression in activated keratinocytes more significantly than free AN2728 and non-targeted nanoparticles. DPNPs also suppressed neutrophil migration by inhibiting keratinocyte-released chemokines. Cytokines/chemokines suppression was involved in the translocation of inhibited p-NF-κB through PDE4 suppression. The actively targeted nanocarriers mitigated psoriasiform symptoms in an IMQ-treated mouse model. DPNPs arrested keratinocyte proliferation and recovered skin barrier function by restraining the production of cytokines/chemokines.

## Supplementary Material

Supplementary figures.

## Figures and Tables

**Table 1:** The size, polydispersity index (PDI) and zeta potential of the nanoparticles

Formulation	Size (nm)	Polydispersity Index (PDI)	Zeta potential (mV)
PLGA	220.07±6.61	0.05±0.01	-26.51±5.65
PLGA+SPC	162.50±1.31	0.11±0.03	-12.12±1.20
PNPs	190.50±2.76	0.12±0.02	-13.25±0.15
DPNPs	229.11±3.81	0.22±0.01	-2.10±0.19

DPNPs, the nanoparticles with antibody conjugation; PLGA, poly(lactic-*co*-glycolic acid); PNPs, the nanoparticles without antibody conjugation; SPC, soybean phosphatidylcholine. Each value represents mean and SEM (*n*=3).
