# Supplementary material for: Monovalent antibody-conjugated lipid-polymer nanohybrids for active targeting to desmoglein 3 of keratinocytes to attenuate psoriasiform inflammation
Source: Theranostics. 2021 Mar 4;11(10):4567–84. doi: 10.7150/thno.56995 (PMC7978323; doi:10.7150/thno.56995)
Supplement: Supplementary file 1 — Supplementary figures. [file thnov11p4567s1.pdf]

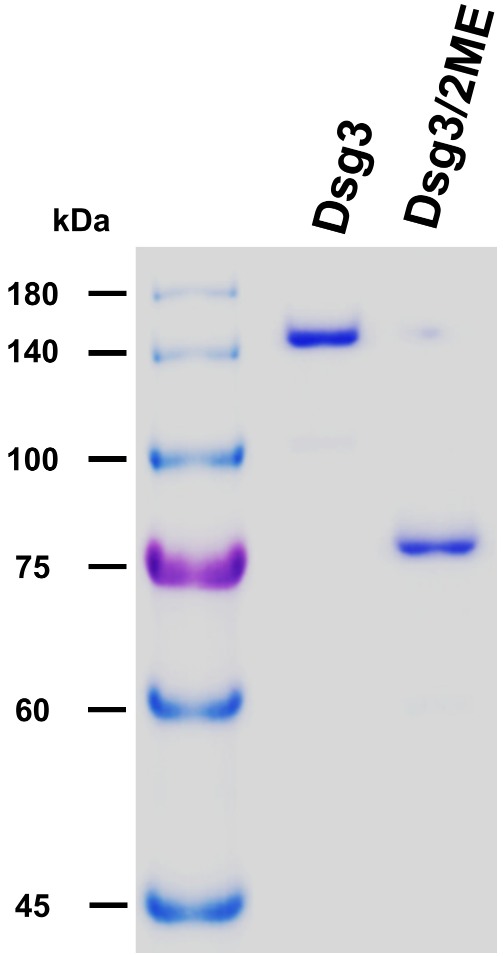

SDS-PAGE analysis of antibody fragmentation

Experimental:
The proteins were separated and analyzed by sodium dodecyl sulfate polyacrylamide gel electrophoresis (SDS-PAGE). SDS-PAGE analysis of antibody fragmentation with the reducing conditions (antibody:β-mercaptoethanol=1:4000) was 8 µL of 0.3 M EDTA, 8 µL of 1 M sodium bicarbonate buffer, and 40 µL of β-mercaptoethanol in 1000 µL PBS solution. After reducing, the antibody was mix with sample buffer then separated using gel electrophoresis to confirm the cleaving conditions for the antibody. Bound antibodies were observed by coomassie blue staining (Invitrogen).

Suppl. Fig. 1

CTL IMQ

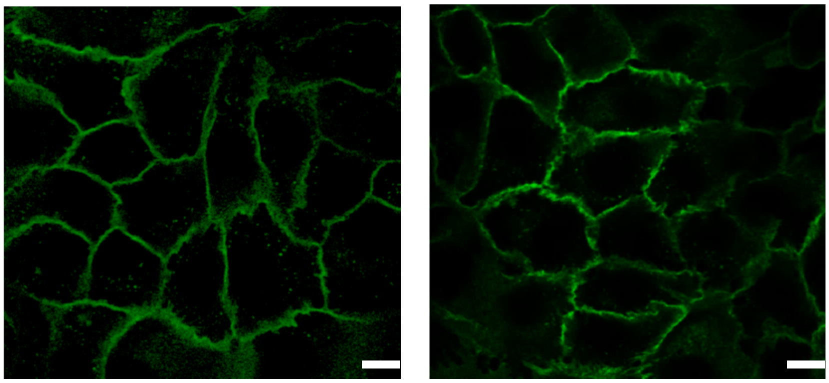

ICC of Dsg3 (HaCaT)

Suppl. Fig. 2

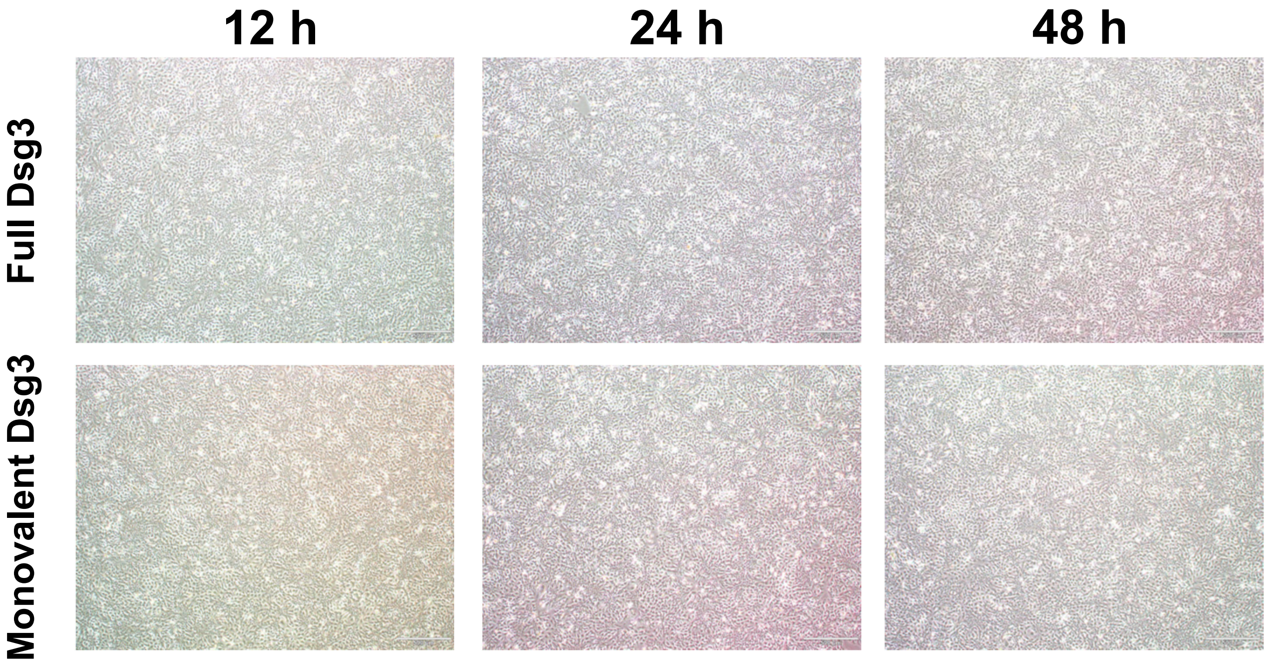

Cell dissociation assay

Experimental:

Keratinocyte dissociation assay was performed with fully confluent HaCaT monolayers on coverslips at a density of 1 x 10^5 cell/mL and treated with 5 µg/mL of full Dsg3 or monovalent Dsg3 antibody. After 12, 24, or 48 h treatment, the cell monolayer was then washed with cold PBS for 5 times before fixation in 4% paraformaldehyde for 30 min. The samples were then processed for Leica DMi8 microscopy.

Suppl. Fig. 3

A

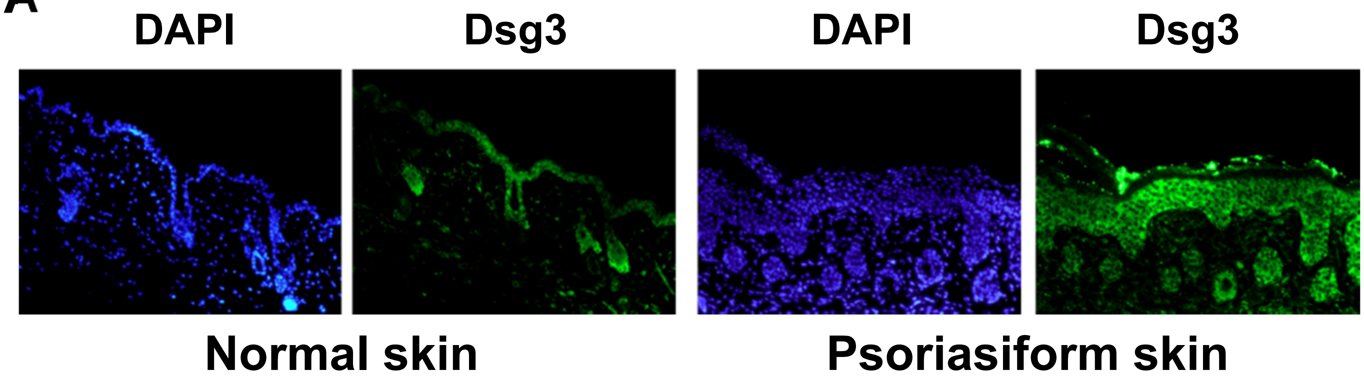

B

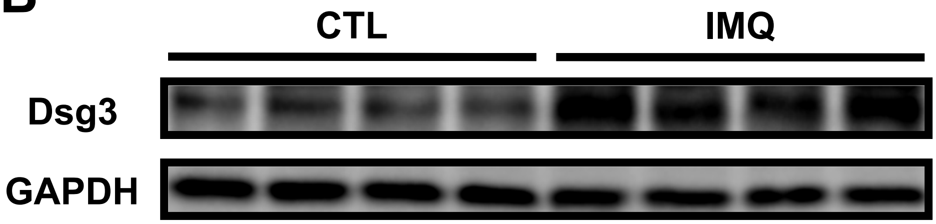

A. IF of Dsg3 (mice skin)
B. Western blot of Dsg3 (mice skin)

Suppl. Fig. 4
